# Ischaemic gastritis and perforation^☆^

**DOI:** 10.1016/j.amsu.2021.103212

**Published:** 2021-12-25

**Authors:** Douglas Chung

**Affiliations:** Campbelltown Hospital, Therry Road, Campbelltown, NSW, 2560, Australia

**Keywords:** Case report, Stomach perforation, Intestinal perforation, Gastritis, Ischaemia

## Abstract

**Introduction:**

Gastric perforation is a common general surgical emergency. Ischaemia of the stomach is uncommon due to its rich vascular supply, and is an uncommon cause of perforation. Minimal literature is available on the topic of ischaemic gastritis, with the few cases available linked to gastric dilation.

**Presentation of case:**

A 52 year old lady presents with a syncopal episode, nausea, vomiting, malaise, and abdominal discomfort. A chest X-ray identified free subdiaphragmatic gas, and her examination revealed a peritonitic abdomen, prompting urgent surgical intervention. She was found to have ischaemic gastritis with multiple perforations along the greater curvature, necessitating a sleeve gastrectomy. Her post-operative course was stormy, requiring significant haemodynamic and respiratory support in the intensive care unit with progressive multi-organ dysfunction. She eventually developed extensive bowel ischaemia, and further management was considered futile. She passed away 13 days post-operatively.

**Clinical discussion:**

The vascular supply of the stomach is rich with collaterals, making ischaemia unlikely. Its occurrence requires either a proximal insult, global ischaemia from pressure, or a systemic coagulopathy. Aside from perforation, it may also present with GI bleeding. Depending on its aetiology, the disease has been managed successfully either conservatively, interventionally, or operatively.

**Conclusion:**

Ischaemic gastritis is uncommon and likely underdiagnosed. Timely recognition of its aetiology early in its course is important, for choosing the appropriate management and to improve patient outcomes. Its management is dependent on the aetiology of the perforation.

## Highlights

• Ischaemic gastritis is uncommon owing to the rich vasculature of the stomach.
• It may present as upper GI bleeding resulting in underdiagnosis.
• Ischaemia requires either a proximal insult, pressure related ischaemia, or coagulopathy.
• Depending on its aetiology, it may be managed conservatively, angriographically, or operatively.
• Delayed presentation significantly worsens outcomes.

## 1 Introduction

Gastric perforation is a relatively common general surgical emergency, with its management in the repertoire of all general surgeons. It usually occurs following as a result of perforation of a peptic ulcer [1]. Due to its rich vascular supply with multiple anastomoses, ischaemia of the stomach is an uncommon but significantly morbid phenomenon. Minimal literature is available on the topic, primarily involving case report, and is mostly linked to gastric dilation [2]. This case report examines one such presentation in line with the SCARE guidelines [3], and undertakes a literature review, with the goal of expanding the available literature on this disease.

## 2 Presentation of case

A 52 year old lady presented to the emergency department following a syncopal episode at home. This was associated with nausea, vomiting of 3 days, and malaise of 3 weeks. She complained of abdominal discomfort when prompted, but denied pain. The patient was haemodynamically unstable throughout her presentation, with a persistent MAP of between 30 and 40 mmHg, which persisted despite fluid resuscitation. She was hypothermic to 35.4 °C, and tachycardic to 120bpm. Despite observations typically seen in extremis, the patient was alert and communicative throughout her pre-operative workup. Her background medical issues include depression for which she was non-compliant with medications; autism; and pilonidal disease. She lived with a supportive mother, and was working and functioning in society prior to her admission.

Due to the nature of her presentation, her management prior to a surgical review included fluid resuscitation and various investigations for syncope, including an ECG and CXR. Subdiaphragmatic air was present, prompting a surgical review. Her abdominal examination revealed a rigid abdomen, with tenderness in the upper abdomen. Her blood tests revealed a pH of 6.93, lactate of 16, renal failure, a white cell count of 17 × 10^9^ cells/L, and a CRP of 74mg/L.

Sepsis secondary to a perforated viscus was suspected, and a decision was made to undertake an urgent laparotomy. She was commenced on intravenous piperacillin/tazobactam, anidulafungin, and a PPI infusion. IV fluid resuscitation was undertaken with minimal effect whilst waiting for theatre availability.

**Fig. 1:**
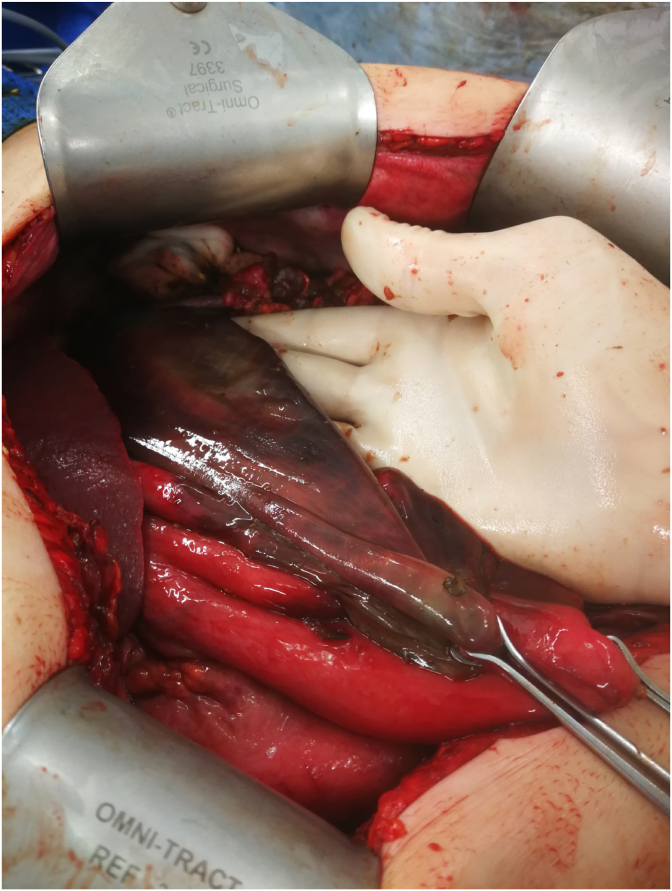
Intraoperative photo.

A laparotomy was performed by the consultant surgeon of the day. Partially digested vegetative material was present throughout all 4 quadrants of the abdomen, particularly in the left upper quadrant, and extricated where possible. A small bowel run and assessment of the colon did not identify any perforations. Removal of vegetative matter in the left upper quadrant revealed significant necrosis of the underlying greater curvature of the stomach (Fig. 1). It had a moth-eaten appearance, and the tissue was friable, tearing easily with minimal instrumentation. Other findings include a spot of ischaemia on the anterior aspect of the stomach. Assistance was requested from a specialist upper gastrointestinal surgeon, who proceeded to perform a partial sleeve gastrectomy of the greater curvature. An intraoperative gastroscopy was undertaken looking for evidence of oesophageal injury, particularly looking for caustic injury, but none was identified. The staple line was oversewn, and an extensive washout was performed with further removal of gastric contents.

Her haemodynamic status improved over the course of the procedure though she continued to require vasopressor and corticosteroid support. She was kept intubated and transferred to the intensive care unit post-operatively. She was commenced on continuous veno-venous haemodialysis due to persistent anuric renal failure. Her hyperlactaemia persisted despite dialysis and continued anti-microbial cover. This progressed to fulminant hepatic failure in and septic cardiomyopathy. 4 days post-operatively, she developed septic emboli to her extremities with ischaemia of various digits. Biochemically and clinically, she appeared to improve day 5 post-operatively with weaning supports with the exception of rising inflammatory markers.

**Fig. 2:**
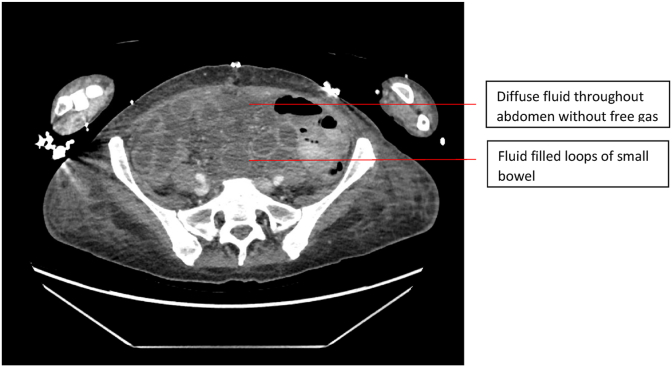
Post-operative CT scan.

A CT abdomen performed day 7 post-operatively revealed diffuse free fluid throughout her abdomen, the significance of which was initially uncertain; and fluid filled loops of small bowel which was attributed to her persistent ileus (Fig. 2). She continued to improve before precipitously deteriorating day 11 post-operatively, with an acute Hb drop and hypotension requiring blood transfusions. Her drains which initially drained haemoserous fluid began draining enteric contents.

Extensive discussions were undertaken between the various teams involved and the patient's family, and a consensus was achieved of an operation to identify the source of leakage and attempt a repair, but with a guarded prognosis, to which the family was understanding. This was performed 13 days after the initial procedure, which found all the small bowel beyond 85cm from the DJ flexure to be necrotic. A septic embolus was thought to be the cause. The situation was deemed non-salvageable, and the procedure was aborted. The patient passed away later that day.

Histopathological examination of the specimen identified florid changes consistent with ischaemic gastritis. Full thickness coagulative necrosis is present histologically, with surrounding areas of patchy mucosal necrosis, extending to the surgical margins. The entire specimen exhibited an acute inflammatory reaction, though not excessive to the point of suggesting an infectious cause. No intravascular thrombi were present to suggest a vasculitic or thrombotic event. Other pertinent negatives absent in the specimen include granulomas, parasites, viral inclusions, dysplasia, or malignancy. While no helicobacter pylori was identified on the specimen, her initial serology was positive.

## 3 Discussion

The vasculature of the stomach is rich with multiple collaterals, deriving its blood supply from the left and right gastroepiploic arteries, left and right gastric arteries, short gastric and gastroduodenal arteries [4], creating a multiple anastamoses and mitigating the risk of local ischaemia.

Scattered case reports suggest a number of causes for ischaemia. Occlusion of the coeliac trunk is one such cause, which may happen in aortic dissection [5]; proximal vagotomy may affect the lesser curve of the stomach, resulting in ischaemic erosion [6]; and acute gastric dilation, either as a result of stretch procedures orgastric distension. Alternatively, multiple vessels have to occlude simultaneously, which is rare outside of a systemic coagulopathy. In our patient's case, the necrosis was along the greater curvature, with patchy involvement of the anterior and posterior aspects of the stomach, suggesting an occlusion of the gastroepiploic arcade bilaterally. This contradicts the histopathological findings which note an absence of thrombi.

Gastric perforation as a result of ischaemia typically presents as the stereotypical acute abdomen, with a haemodynamically unstable patient sporting a rigid abdomen. Less commonly, gastric ischaemia may present as upper gastrointestinal bleeding, with reports of patients developing malaena. Patients stable enough to undergo a gastroscopy typically demonstrate mucosal congestion, erythema and possible ulceration [7], with ischaemic gastric tissue identified on histology [8]. Gastric pneumoatosis is a worrying sign warranting urgent attention. Most lower GI bleeding is typically managed with watch and wait strategies followed by interval endoscopies, at which point the initial insult may have resolved. This has been thought to result in under-recognition of this clinical syndrome.

The management of gastric ischaemia depends on its presentation. Common measures which should be undertaken in the emergency department include utilisation nasogastric tube placement, fluid resuscitation, acid reduction, and antimicrobial agents in the appropriate setting [9]. Gastric erosion and bleeding is typically the result of systemic abberations in circulation, and lends itself to conservative management. A case series on coeliac territory ischaemia [10] in utilised gastric revascularisation with various bypasses to restore circulation to the stomach with good effect, which include aortohepatic, renohepatic, and retrograde ileosuperior bypasses. Coeliac artery angioplasty was also utilised in another case with rapid healing afterwards. Gastroduodenal perforation may occasionally be managed conservatively in small localised perforations. Depending on the degree of contamination and initial cause, the more common approach is operative management with a Graham patch. In our patient, due to the size of the perforation, we elected to perform a partial sleeve gastrectomy to salvage the remaining portion of stomach. Timely recognition of this disease is vital, with mortality reaching 24% at 6 months following the diagnosis [7].

Unfortunately, the patient's delayed presentation likely resulted in the poor outcome patient experienced, exacerbated by her suffering from autism. The patient's family was contacted, and on discussion were in agreement that this was the primary factor. Earlier recognition of this condition resulted in a more favourable outcome.

In retrospect, this patient's pathology is likely a combination of local and systemic factors. The initial insult is likely a gastric perforation from a peptic ulcer. This progressed to septic shock as she delayed her presentation, resulting in splanchnic hypoperfusion. This occurred alongside local inflammation inducing hypoperfusion around the site of perforation to produce the localised region of gastric ischaemia identified intraoperatively.

## 4 Conclusion

Ischaemic gastritis is an uncommon and likely underdiagnosed entity with varying presentations. However, there is significant associated mortality. As such, recognition of its aetiology is important in choosing the appropriate management. Depending on its nature, this disease may be managed conservatively, interventionally, or operatively. Early recognition and management is vital in management.

## Sources of funding

This research did not receive any specific grant from funding agencies in the public, commercial, or not-for-profit sectors.

## Provenance and peer review

Not commissioned, externally peer-reviewed.

## Ethical approval

Written informed consent was obtained from the patient for publication of this case report and accompanying images, in line with local ethical approval requirements. No other requirements were stipulated.

## Consent

Written informed consent was obtained from the patient for publication of this case report and accompanying images.

## Author contribution

Douglas Chung - Corresponding author.

- Case report design
- Data collection
- Data interpretation
- Writing the paper

## Registration of research studies

N/A.

## Guarantor

The corresponding author is the guarantor of this manuscript.

## Declaration of competing interest

No conflicts of interest were identified in the writing of this case report.
